# Psychological Determinants of Blood Donation During the COVID-19 Pandemic in Hungary

**DOI:** 10.1007/s12288-024-01867-y

**Published:** 2024-09-06

**Authors:** László Dorner, Georgina Csordás

**Affiliations:** 1 Department of School Psychology, Istitute of Psychology, Eszterházy Károly Catholic University, Eszterházy tér 1, Eger, 3300 Hungary; 2 Department of Developmental and Educational Psychology, Eszterházy Károly Catholic University, Eger, Hungary

**Keywords:** COVID-19, Blood donor, Motivation, Habit and identity motivation, Self-efficacy

## Abstract

The COVID-19 pandemic had a considerable impact on blood donations worldwide. The present study aims to empirically investigate the determinants of willingness to donate blood during the COVID-19 pandemic in Hungary. *Methods* Our study was carried out on 418 whole blood donors (286 women, 68.4%) using data collection from the online and paper self-report questionnaire data collection. We evaluated the association between motivation, self-efficacy, and technical and emotional barriers to donation. *Results* The correlational analysis showed that the number of donations during the COVID-19 pandemic was positively associated with the total number of donations; habit and identity motivation; and blood donation self-efficacy. In contrast, a negative association has been found with perceived barriers during the pandemic. The path model confirmed that the number of donations during the pandemic can be explained by the donors' habit and identity motivation facilitated by the experienced blood donation self-efficacy. The number of total donations also predicted the donations during COVID-19. *Conclusion* These findings suggest that high donor self-efficacy is associated with habitual donation and the need to reinforce donor identity, which may again positively influence the number of donations. These factors contribute to the continued willingness to donate and result in a stable blood supply even during a health crisis or other societal challenges. Our research fits into a series of studies that draw the attention of blood donation professionals to the importance of identifying and maintaining a motivational background, increasing self-efficacy, and removing barriers to blood donation.

**Supplementary Information:**

The online version contains supplementary material available at 10.1007/s12288-024-01867-y.

## Introduction

Voluntary blood donation is an essential social contribution to the community, as human blood is currently scientifically unsubstitutable and the importance of non-remunerated blood donors to ensure a safe and sustainable blood supply for routine surgeries and emergencies in every country is indisputable [1].

The outbreak of the SARS-COV-2 pandemic, its international spread, and the associated epidemiological measures (e.g. temporary exclusion from the donation of blood of those suspected of being infected with the new type of coronavirus) have challenged the transfusion care system [2, 3] resulting in a worldwide decline in the number of blood donation events (e.g. due to the loss of blood donation sites at schools and places of employment) [4]. A postponement of elective surgeries and the emergence of COVID-19 among donors has required constant replanning of the supply and demand for blood products [5].

In Hungary, blood donation is voluntary and unpaid (donors receive a food voucher of 1000 HUF equal to 2.2 GBP as a "calorie replacement" upon donation), and annually more than 390 000 units of blood products are needed and collected [6]. Due to restrictions, a significant part of mobile blood donation events was already cancelled during the first months (March–May, 2020) of the pandemic; thus, the number of institutional donations decreased, resulting in a temporary decrease in blood supply [7, 8]. Effective recruitment efforts were required to maintain the willingness of experienced donors and to attract new donors in this ever-changing situation. In both 2020 (329,192) and 2021 (332,970), Hungary had 50,000 fewer units of blood donated per year than in the period preceding the COVID-19 pandemic (13% decrease compared to 376,744 units in 2019). The numbers for 2022 (343,643) and 2023 (355,455) indicate that blood donations show a slight increase again [9]. The age distribution of donors is also worth a brief overview (see Supplement 1).

Investigating the motivations for voluntary blood donation has long been a focus of research [10, 11] with numerous studies highlighting the motivations of blood donors to engage, sustain, or withdraw from the activity and the help blood donors offer in emergencies. Most blood donors cite altruism, civic responsibility, and prosocial intentions as their primary motivation [12–14], but social [15] and other self-regarding motives are also important [16]. Overall, each donor exhibits a unique interplay between self-interest and altruism, which may vary depending on personality traits, age, life circumstances, as well as moral commitments and social influences [13, 17, 18].

Turning to the COVID-19 trends, recent research [3, 19–21] reported altruism and the desire to fight against the pandemic as the main motivations for blood donation.

A study of the first months after the COVID outbreak showed that although the risk of infection during donation was relatively low, donors who expected a high risk of infection were much less likely to donate blood [3]. Tran et al. [22] highlight that during the pandemic, fearing the risk of COVID-19 infection (53.5%) was identified as key barrier for blood donors. Furthermore, those who adhered to the COVID guidelines were less likely to donate blood [3].

The literature on blood donor motivation has demonstrated the importance of self-efficacy as a predictor of donation intention [23–26]. Blood donation self-efficacy is related to the extent to which individuals are confident in their ability to give blood [10]. While Masser et al. [27] showed that intention to donate during the pandemic was positively predicted by self-efficacy, Veseli et al. [28] showed that self-efficacy of active donors increased, and inactive donors’ self-efficacy decreased compared to pre-pandemic; the pandemic effect on reported donor motivations is, therefore, less negative for active donors. As highlighted, although the motivational background of voluntary donation is well researched, the motivational background of donation under COVID-19 and its inhibiting technical (e.g. confinement, the distance of blood banks), health-related (more side effects of blood donation) and emotional (e.g. fear of being infected, increased stress level) factors in Hungary are less understood. The present research aims to fill this gap including the creation and testing of a model underlying the factors behind blood donations during the pandemic. In this model, perceived blood donation self-efficacy enhances the habit and identity blood donation motivation and the number of total donations, which predict positively the number of donations during the pandemic.

## Materials and Methods

### Participants

The data collection was completed as a non-probability sampling in cooperation with the Hungarian Red Cross. Two criteria were defined for including participants: one for the respondent to be of the legal age of 18 and one for the active whole blood donor status. The final sample size was 418 (31.6% were male and 68.4% were female) and heterogeneous both in terms of demographic background (18 counties) and age (18–68 years, M = 41.91; SD = 10.61), with the total number of donations (range = 1–163, M = 20.57) and the number of donations during COVID-19 (range = 0–9, M = 2.89). By type of residence, 17.9% of the participants lived in a capital city, 29.9% in a regional centre or larger town, 29.4% in a smaller town, and 22.7% in a village. Nearly half of the respondents were married and a quarter of them were currently in a relationship, 17.9% were single and 6.7% were divorced or separated. The majority (60.9%) of the sample had a tertiary and roughly a third of them had a secondary education level. 42.6% of the sample reported being religious. The descriptive data can be seen in Supplement 2.

### Procedure and Measures

Before the data collection, the authors of this study obtained the approval of the Ethics Review Committee for Research at Eszterházy Károly Catholic University (RK/995/2021). In the first wave of data collection (August-November 2021), the paper-and-pencil survey was administered to a random sample of blood donors in each of the Hungarian regions allowing for a broad sample of age and demographic characteristics to be included. The data collection was conducted with the help of volunteer coordinators of the Hungarian Red Cross regional centres. In the meantime, an on-line version of the survey was also available. In this phase, a total number of 219 donors participated. The second wave of data collection was carried out online via the National Blood Service's newsletter, website and Facebook page between March and April 2022 (N = 199). The on-line data was collected through the UNIPOLL system in both phases. Before completion, prospective participants were provided with written information highlighting that their participation in the study was voluntary and anonymous and that the data obtained from the survey would be treated confidentially. Afterwards, the participants gave their informed consent to participate in the research.

The preliminary analyses were conducted using SPSS Version 26, and the path modelling analysis was conducted with JASP Version 0.19.0 [29]. We used Pearson’s correlation analysis to investigate the association between motivational and COVID-related factors, and intention to donate in the next 6 months. Path model analysis was conducted by the maximum likelihood estimation using the bootstrapping method. We evaluated the fit of the model with the following indices: χ^2^, RMSEA, SRMR, and CFI.

The survey contained the following parts:

#### Blood Donation History

The questions were selected on the basis of previous similar studies [30]. Respondents were asked if they had given whole blood before and, if so, how many times. The number of donations since the start of the COVID-19 pandemic were evaluated (11 March 2020), and the total number of their donations so far (See Supplement 3).

#### COVID-19-Related Concerns and Perceived Barriers

The questionnaire was designed in the present research using similar studies [3] and advice from experts working in the blood transfusion service. In this scale, eight items were used to examine the extent to which COVID-19 was associated with barriers like *fear and safety* (e.g., "I was afraid of contracting COVID at the blood donation site";”I did not feel safe to donate blood”), *changed life circumstances and perceived inconveniences* (e.g., “confinement was a barrier to blood donation”, “I have suffered personal losses'', “My general stress level increased”, My general activity level decreased”, “I experienced more side effects from donating blood than before the pandemic”; “It was harder to find a suitable blood donation venue than before the pandemic”). As the next step, a cumulative scale was created. The scale has a good reliability index (α = 0.869).

#### Hungarian Blood Donor Motivation Scale

The validated questionnaire [31] measures the motivational background of blood donors with twenty items across four factors. The first factor (*Self-growth*) contains eight items that are linked to the " personal gains" associated with donating blood, in particular, good feelings (sense of importance), overcoming personal problems, making new relationships, and health-related aspects (e.g. "Donating blood helps to overcome my problems"). The second factor (*Habit and Identity*) contains three items that highlight the habit- and identity-forming nature of blood donation (e.g. “It has become a habit for me to give blood, so I don't think about the reasons for it”). The third factor *(Altruism and Prosocial Behavior*) contains four items, which refer to the importance of helping others, compassion, caring, and prosocial values (e.g. "I donate blood because I feel compassion for those needing blood products"). The fourth factor is *Social Reference*, which contains five items with the positive influence of close contacts on blood donation, for example, whether the friends-acquaintances would like the person to donate blood (e.g. “People close to me want me to give blood”). The scale has a good reliability index (α = 0.826).

#### Blood Donation Self-Efficacy Scale

The four self-efficacy items (edited by the authors in this study) assessed an individual’s belief of their confidence and perceived ability to donate blood successfully (For example; “I believe in my capability to donate blood”; “I consider myself as able to be a blood donor as long as my health allows it”) on a five-point Likert scale. The scale achieved good internal consistency validity with a high Cronbach’s α of 0.82 in this study. The factor structure indicated good model fit during the confirmatory factor analysis (*χ*^*2*^ (2) = 3.26, *p* = 0.20, *χ*^*2*^/df = 1.63, RMSEA = 0.05, SRMR = 0.02, CFI = 0.99, TLI = 0.99).

#### Intention to Give Blood in the Next Six Months

The return of intention to donate blood was measured with a three-item scale. It assesses the willingness of the respondents to donate blood within the next 6 months. The scale measures the extent to which individuals intend to donate blood again within the next six months. It consisted of three items (“I plan to donate blood within the next 6 months”; “I want to donate blood during the next 6 months”; and “I intend to donate blood within the next 6 months”) with a 5-point Likert scale. This scale achieved good internal consistency validity with a high Cronbach’s α of 0.96 in this study.

## Results

### Correlational Analysis

As Table 1 shows, the total number of donations showed a significantly positive correlation (*r* = 0.392 *p* < 0.01) with the number of donations during COVID-19. Habit and identity turned out to be the only motivational factor associated with the number of donations during the pandemic (*r* = 0.338 *p* < 0.01) and with the number of total donations alike (*r* = 0.274 *p* < 0.01); in addition, COVID-related barriers were negatively (*r* = 0.268* p* < 0.01), while blood donation self-efficacy was positively (*r* = 0.280 *p* < 0.01) related to the number of donations during the pandemic. Blood donation self-efficacy showed the strongest positive relation with habit and identity motivation (*r* = 0.478 *p* < 0.01). The intention to give blood in the next 6 months showed the strongest positive correlation with blood donation self-efficacy *(r* = 0.467 *p* < 0.01) and with habit and identity motivation (*r* = 0.337 *p* < 0.01), while showing a weaker positive correlation with altruism motivation (*r* = 0.171 *p* < 0.01), and a negative correlation with COVID-related barriers (*r* = −0.193* p* < 0.01). COVID-related barriers also shows a negative correlation with habit and identity motivation (*r* = −0.142* p* < 0.01) and blood donation self-efficacy (*r* = −0.141* p* < 0.01).

**Table 1:** Bivariate relationship among included variables

Variables	1	2	3	4	5	6	7	8
1. Number of total donations								
2. Number of donations during COVID-19	.392**							
3. COVID-related barriers	−.195**	−.268**						
4. Self-growth motivation	.072	.042	.068					
5. Habit and identity motivation	.274**	.338**	−.142**	.480**				
6. Altruism and prosocial motivation	.017	−.022	−.027	.429**	.404**			
7. Social reference motivation	.095	−.060	.015	.404**	.256**	.344**		
8. Blood donation self-efficacy	.210**	.280**	−.141**	.177**	.478**	.251**	.137**	
9. Intention to give blood in the next 6 months	.126*	.233**	−.193**	.082	.337**	.171**	.041	.467**

*p < .05, **p < .01

### Path Model

In the model, all of the proposed direct and indirect paths were significant (see Table 2). The model fit was adequate according to the CFI (= 0.98) and SRMR (= 0.20) values. The *χ*^*2*^ (= 6.11, df = 1) and RMSEA (= 0.11, CI = 0.04–0.20) indices indicated poor fit; however, the *χ*^*2*^ test is sensitive to sample size, and the RMSEA index could be misleading in models with low degrees of freedom. Considering the latter and the low values of the modification indices (the largest value was 5.57), we decided to accept the model.

**Table 2:** Results of the path analysis

Predictor	Outcome	Standardized regression coefficient	*p*
Blood donation self-efficacy	Habit and identity motivation	.48	< .001
Blood donation self-efficacy	Number of total donations	.21	< .001
Number of total donations	Number of donations during COVID-19	.32	< .001
Habit and identity motivation	Number of donations during COVID-19	.26	< .001
Path		Standardized regression coefficient	*p*
Blood donation self-efficacy—Habit and identity motivation—Number of donations during COVID-19		.74	< .001
Blood donation self-efficacy—Number of total donations—Number of donations during COVID-19		.07	< .001
		R^2^	
Habit and identity motivation		.23	
Number of total donations		.04	
Number of donations during COVID-19		.21	

**Fig. 1:**
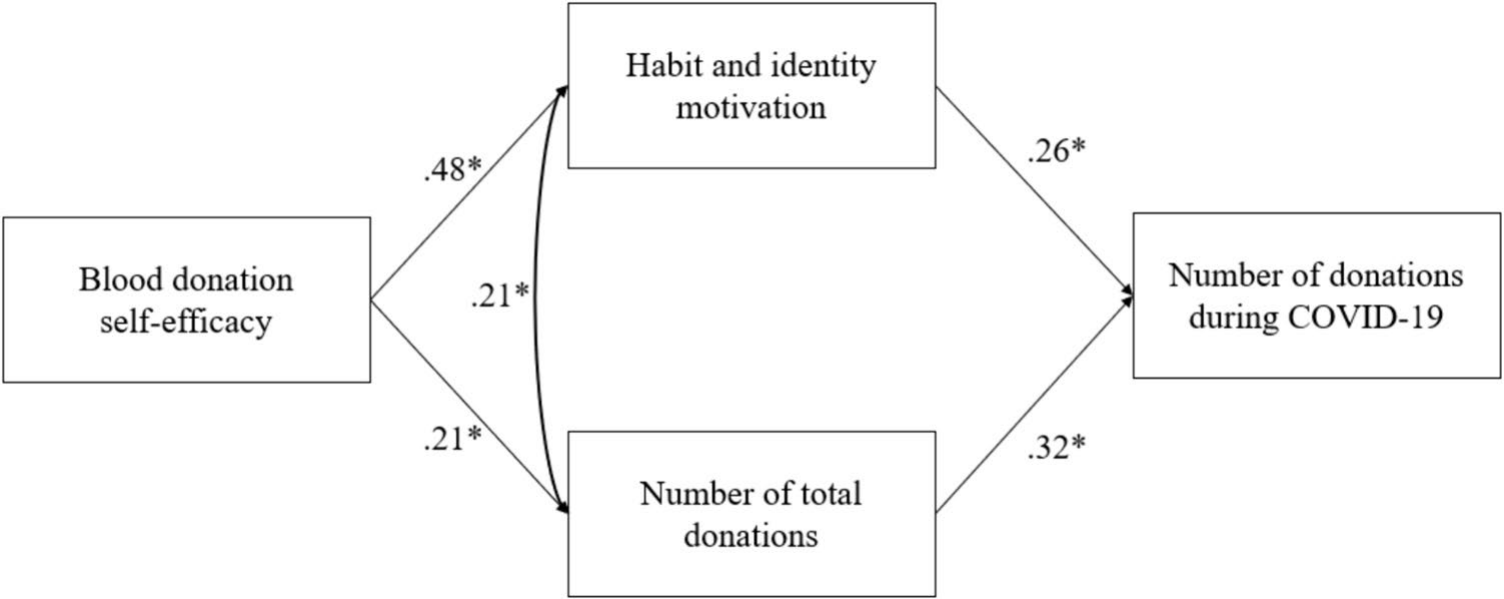
Path model. *Notes* standardized coefficients are included in the figure. *p < .001

According to the model, the number of donations during the pandemic can be explained by the donors' habit and identity motivation facilitated by the experienced blood donation self-efficacy. The number of total donations, as a control variable, also predicted the donations during COVID-19 significantly, but with a small coefficient (see Fig. 1).

## Discussion

The COVID-19 pandemic presented unprecedented challenges to blood donation services around the world. The present study focused on the psychological determinants of blood donation in Hungary and the role of the deterrents during this period. In terms of donor motivation, in contrast with some former studies [3, 19–21, 31], not altruism, but habit and identity motivation and total number of donations proved to be the key factors for predicting the number of donations during the pandemic. This result can be interpreted in lieu of the blood donation tendencies. Matusovits et al. [8] showed that in Hungary the response of blood donors showed considerable variability during the severe restrictions: the most committed and active (≥ 10) donors were able to relatively maintain their donation activities, but those with a medium number (1–9) of previous donations showed profoundly larger decreases in numbers, while first-time donors having been the most pandemic sensitive. Regarding the predictive role of total number of blood donations, our results are in line with the results of Chandler et al. [3] who found that previous blood donation activity in 2 years before COVID-19 was a strong predictor of donating during the crisis period. Furthermore, Veseli et al. [28] stated that during the pandemic, both active and inactive donors had felt less responsible and less morally obliged to donate, resulting in an overall negative pandemic effect on blood donation intentions. This trend may partially explain the findings that people with higher self-reported altruism and prosocial motivation were less responsive to the COVID crisis compared to those for whom donating blood has become a habit and part of their identity. Steele et al. [14] have already noted that although most blood donors have high levels of prosocial characteristics (e.g., altruism), this is not the strongest determinant of the frequency of donation. The findings also highlight the role of self-efficacy as a “bridge” from eligibility to actual donor status throughout the whole blood donor career: self-efficacy is related to the number of donations both in the lifetime and during the pandemic, which corroborates with the results of Li et al. [25] and Bednall et al. [10] who found that experienced donors were more strongly influenced by self-efficacy and with the results of Masser et al. [27]. Still, in our path model, habit and identity motivation was found to be the more accurate predictor. Blood donor self-identity, which can be developed as a result of ongoing donations, meeting friends through donation, anticipating future donations, and describing oneself as a donor [32] is also of paramount importance for a stable future blood supply. Our results are in line with both quantitative [26, 33, 34] and qualitative [35] research that found COVID-19-related obstacles (e.g. fear of infection, anxiety) to be deterrents to donating blood.

## Conclusion

Based on the survey results, we propose that blood donor organizations consider launching specific campaigns that focus not only on donors' altruistic motivation but *building their donor identity* ("Blood donation is a lifesaving habit", „Come and donate blood again” or giving blood donors small gifts), *community sense* (“Be part of the blood donor community”) and *self-efficacy* ("You are capable of giving blood") even in times of crisis, continue to communicate the safety of blood donation, stress that barriers to blood donation can be overcome and the presence of professional help if needed.

## Limitations of the Study

The study was the first to measure the motivational and situational factors behind blood donation during the COVID-19 pandemic in Hungary. The first limitation is a non-representative sample of blood donors in Hungary. In addition, the results show limitations due to self-reported motivations. Furthermore, due to the difficulty in accessing participants during the pandemic, a two-phase data collection approach was used: first, one paper-based and online, and later an online data collection. At the same time, recall bias may have been present, which could have limited the interpretation of the results. Supposedly, more conclusive data would have been derived from multiple inquiries of the same donors over the COVID period. Future research should mitigate these shortcomings.

## Supplementary Information

Below is the link to the electronic supplementary material.

Supplementary file1 (DOCX 45 kb)

## Data Availability

The datasets used and/or analysed during the current study are available from the corresponding author upon reasonable request.
