# Supplementary material for: Psychological Determinants of Blood Donation During the COVID-19 Pandemic in Hungary
Source: Indian J Hematol Blood Transfus. 2024 Sep 6;41(2):363–9. doi: 10.1007/s12288-024-01867-y (PMC11992318; doi:10.1007/s12288-024-01867-y)
Supplement: Supplementary file 1 — Supplementary file1 (DOCX 45 kb) [file 12288_2024_1867_MOESM1_ESM.docx]

**Supplement 1.** *Distribution of total donors in Hungary, 2019-2023*

If we see the blood donor characteristics in Hungary, the 18-25 age group already represents a significant proportion, while the strongest base is represented by the 41-45 and 46-50 age groups, with the profile showing a steadily ageing trend over the years.


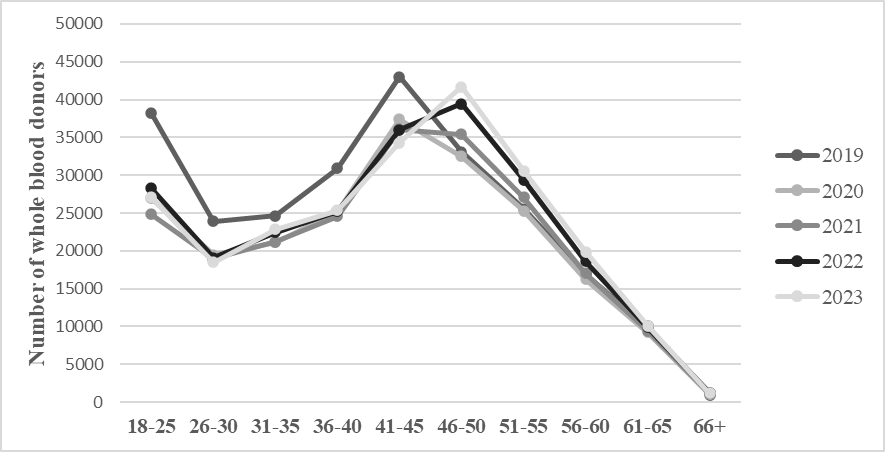


Distribution of total donors in Hungary by age groups between 2019 and 2023. Edited by the authors based on the data service of Nagy [12]

**Supplement 2.** *Demographic variables in the sample*

| **Variable** | **Category** | | **Number of participants (%)** | | | |  |
| --- | --- | --- | --- | --- | --- | --- | --- |
| Sex |  | Female | |  | 286 (68.4%) |  | |
|  |  | Male | |  | 132 (31.6%) |  | |
| Age |  | 18-24 | |  | 30 (7.2%) |  | |
|  |  | 25-34 | |  | 70 (16.7%) |  | |
|  |  | 35-44 | |  | 136 (29.9%) |  | |
|  |  | 45-54 | |  | 125 (23.5%) |  | |
|  |  | 55-64 | |  | 51 (11.3%) |  | |
|  |  | 65+ | |  | 6 (1.4%) |  | |
| Education |  | Primary | |  | 6 (1.4%) |  | |
|  |  | Secondary | |  | 157 (37.7%) |  | |
|  |  | Tertiary | |  | 255 (60.9%) |  | |
| Marital status |  | Married | |  | 193 (46.2%) |  | |
|  |  | In a relationship | |  | 106 (25.4%) |  | |
|  |  | Single | |  | 75 (17.9 %) |  | |
|  |  | Divorced or separated | |  | 28 (6.7%) |  | |
|  |  | Widowed | |  | 11 (2.6%) |  | |

**Supplement 3:** *Blood* *donation history of the respondents*

| Number of total blood donations |  |  |  |  |
| --- | --- | --- | --- | --- |
|  |  | 1-10 |  | 177 (43.9%) |
|  |  | 11-20 |  | 95 (23%) |
|  |  | 21-40 |  | 82 (17.8%) |
|  |  | 41-60 |  | 30 (6.7%) |
|  |  | 61+ |  | 26 (6.3%) |
| Number of donations during COVID-19 |  | 0-2 |  | 205 (52.2%) |
|  |  | 3-5 |  | 142 (36.1%) |
|  |  | 6-9 |  | 46 (11.7%) |
